# Analysis of *Schistosoma mansoni *genes shared with Deuterostomia and with possible roles in host interactions

**DOI:** 10.1186/1471-2164-8-407

**Published:** 2007-11-08

**Authors:** Thiago M Venancio, Ricardo DeMarco, Giulliana T Almeida, Katia C Oliveira, João C Setubal, Sergio Verjovski-Almeida

**Affiliations:** 1 Laboratory of Bioinformatics; Departamento de Bioquímica, Instituto de Química, Universidade de São Paulo, 05508-900 São Paulo, SP, Brazil.; 2 Laboratory of Gene Expression in Eukaryotes; Departamento de Bioquímica, Instituto de Química, Universidade de São Paulo, 05508-900 São Paulo, SP, Brazil.; 3 Virginia Bioinformatics Institute, Virginia Polytechnic Institute and State University, Blacksburg, VA, USA.

## Abstract

**Background:**

*Schistosoma mansoni *is a blood helminth parasite that causes schistosomiasis, a disease that affects 200 million people in the world. Many orthologs of known mammalian genes have been discovered in this parasite and evidence is accumulating that some of these genes encode proteins linked to signaling pathways in the parasite that appear to be involved with growth or development, suggesting a complex co-evolutionary process.

**Results:**

In this work we found 427 genes conserved in the Deuterostomia group that have orthologs in *S. mansoni *and no members in any nematodes and insects so far sequenced. Among these genes we have identified Insulin Induced Gene (INSIG), Interferon Regulatory Factor (IRF) and vasohibin orthologs, known to be involved in mammals in mevalonate metabolism, immune response and angiogenesis control, respectively. We have chosen these three genes for a more detailed characterization, which included extension of their cloned messages to obtain full-length sequences. Interestingly, SmINSIG showed a 10-fold higher expression in adult females as opposed to males, in accordance with its possible role in regulating egg production. SmIRF has a DNA binding domain, a tryptophan-rich N-terminal region and several predicted phosphorylation sites, usually important for IRF activity. Fourteen different alternatively spliced forms of the *S. mansoni *vasohibin (SmVASL) gene were detected that encode seven different protein isoforms including one with a complete C-terminal end, and other isoforms with shorter C-terminal portions. Using *S. mansoni *homologs, we have employed a parsimonious rationale to compute the total gene losses/gains in nematodes, arthropods and deuterostomes under either the Coelomata or the Ecdysozoa evolutionary hypotheses; our results show a lower losses/gains number under the latter hypothesis.

**Conclusion:**

The genes discussed which are conserved between *S. mansoni *and deuterostomes, probably have an ancient origin and were lost in Ecdysozoa, being still present in Lophotrochozoa. Given their known functions in Deuterostomia, it is possible that some of them have been co-opted to perform functions related (directly or indirectly) to host adaptation or interaction with host signaling processes.

## Background

*Schistosoma mansoni *is a digenetic platyhelminth trematode and is one of the major causative agents of Schistosomiasis [1], a disease that affects 200 million infected individuals and an additional 500–600 million are at risk [2]. Schistosomiasis is a neglected disease occurring primarily in impoverished urban areas of developing countries and is considered not only a consequence of poverty, but also a poverty-promoting condition in the affected populations [3]. Parasite eggs laid in the hepatic portal vasculature are the principal cause of morbidity, and the ensuing pathology may prove fatal [4]. Inhibition of protein tyrosine kinases has been shown to interfere with egg production and suggested as a novel strategy to combat schistosomiasis [5]. Eggs are highly immunogenic and capable of inducing potent Th responses [6]. Protective immune mechanisms in humans that might form the basis for a vaccine have proven difficult to characterize [7], owing to effective immune evasion by the parasites. Active interactions with the host play an important role in the parasite immune evasion process, through detection of hormones and other host signaling molecules [8].

Two large-scale independent efforts have obtained significant numbers of transcriptome sequences from *S. mansoni *[9] and *S. japonicum *[10], and the draft of the genome sequence of *S. mansoni *is currently being assembled [11]. Recently, large-scale transcriptome sequencing of the planarian *Schmidtea mediterranea *has provided molecular information about a free-living platyhelminth [12]. These datasets are the first large repository of mRNA sequences for platyhelminth organisms and have therefore provided insights into the evolution and molecular biology of these organisms, as well as help in understanding adaptation to parasitism of *S. mansoni *and identification of gene products to be exploited as novel drug targets and vaccine candidates. Using primarily the data generated by the *S. mansoni *EST Genome Project [9] here we present a detailed investigation of certain *S. mansoni *genes that we believe provide important insights into the biology of this organism.

**Figure 1:**
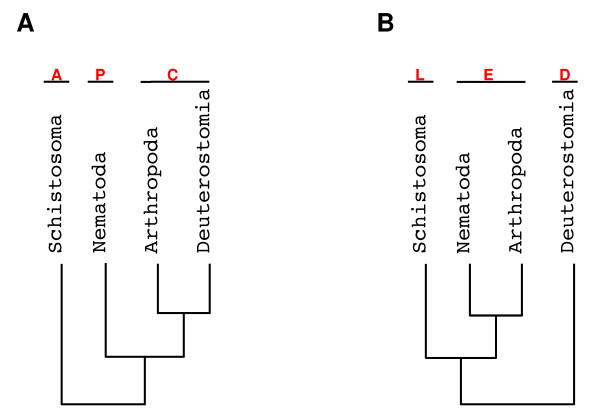
**Schematic representation of the two main hypotheses for the relationships between animal phyla**. **A **– Acoelomata-Pseudocoelomata-Coelomata hypothesis; **B **– Lophotrocozoa-Ecdysozoa-Deuterostomia hypothesis. This scheme is illustrative and branch lengths do not reflect evolutionary distances.

The schistosoma genus is part of the platyhelminth phylum, which has been traditionally regarded as one of the first diverging phyla of the bilaterian group in the acoelomate-pseudoceolomate-celomate (APC) theory (Figure 1A), which groups bilaterally symmetrical animals based on the presence of coelom (a body cavity lined by an epithelium derived from mesenchyme, e.g. human pleural cavity) [13,14]. This view is based on a gradualist scenario in which the first bilaterian ancestral was acoelomate and some of its descendants developed coelomic cavities originating the various coelomate phyla. Recent analysis of molecular data and embryonic development suggested that platyhelminths are not in the basal position of bilateria, but are derived from an ancestral coelomate [15-17]. This new phylogeny classifies bilaterian animals in *deuterostomes *(the first opening, the blastopore, becomes the anus) and *protostomes *(the first opening becomes the mouth). The Deuterostomia group includes all chordates and echinoderms. Protostomes are further divided in Lophotrochozoa (animals with a feeding structure called lophophore; e.g. platyhelminths, annelids and mollusks) and Ecdysozoa (animals that undergo ecdysis or moulting, e.g. insects and nematodes) [15-17]. This hypothesis is named LED (Lophotrochozoa-Ecdysozoa-Deuterostomia) (see Figure 1B).

Wolf et al. [18], analyzing the evolutionary relationships between animal phyla using the predicted genes of fully sequenced organisms, obtained strong statistical support for the APC hypothesis (Figure 1A). Philippe et al. [15] analyzing a few genes from multiple species (representing several taxa) provided support for the LED hypothesis (Figure 1B). Philippe and collaborators [15,16,19] pointed out that despite the strong statistical support argued by Wolf et al. [18], long-branch attraction artifacts might have affected the results.

The LED hypothesis has been widely supported in the last few years by innovative approaches, such as intron conservation analysis [20] and a whole genome phylogeny analysis excluding *C. elegans *fast-evolving sequence genes [21]. However, there is also a recent phylogenetic analysis using large scale molecular data that supports the APC phylogeny [22]. Therefore, metazoan evolution is still an open question in phylogenetics.

## Results and Discussion

Our goal was to identify *S. mansoni *genes in the large transcriptome sequence collection that are conserved among platyhelminths and Deuterostomia, thus providing new insights into *S. mansoni *biology. The two major evolutionary theories of metazoans provided a framework for this identification as follows. We selected three main taxonomic groups to focus our work on: Nematoda, Arthropoda and Deuterostomia. These groups are monophyletic under both the APC and LED hypotheses (Figure 1A and 1B). We selected a total of 6,504 *S. mansoni *genes for which we found orthologs in at least one organism representing at least one of our focus groups. We then determined the presence/absence of these genes in large merged datasets (e.g. all nematodes, all arthropods; see Methods for details). Of the selected 6,504 *S. mansoni *genes, we found that 4,244 were present in at least one organism from each of the other three clades, thus not contributing to losses/gains at the clade level (Table 1). All the other 2,260 genes in our analysis (Table 1 and Additional file 1) contributed to gene gain/loss event counts (Table 1). Overall, there were 3,123 gain/loss events under the APC hypothesis and 2,757 events under the LED hypothesis. The excess of 366 events under the APC hypothesis is statistically significant (p-value = 2.2 × 10^-16^), which favors the LED theory under the parsimony rationale [19].

**Table 1:** Number of postulated gain/loss events in comparing *S. mansoni *genes with those present in nematodes, arthropods and deuterostomes

	Cases*				**# of genes**	**# of Loss/Gain events under each theory**	
	Sm	Nem	Arthr	Deut		**APC**	**LED**

Group 1	1	1	1	1	4,244	0	0
Group 2	1	0	0	1	427	2 × 427 = 854	1 × 427 = 427
Group 3	1	0	1	0	436	2 × 436 = 872	2 × 436 = 872
Group 4	1	1	0	0	61	1 × 61 = 61	2 × 61 = 122
Group 5	1	1	1	0	289	1 × 289 = 289	1 × 289 = 289
Group 6	1	0	1	1	998	1 × 998 = 998	1 × 998 = 998
Group 7	1	1	0	1	49	1 × 49 = 49	1 × 49 = 49

**TOTAL**					6,504	3,123	2,757
**Excess losses/gains (APC – LED)**						366	
Significance (p-value)**						2.2 × 10^-16^	

* *S. mansoni *(Sm) genes were compared to genes from all organisms of the following clades: Nematodes (Nem), Arthropods (Arthr) and Deuterostomes (Deut). Each group represents one of the possible cases: when *S. mansoni *genes were present in at least one organism of the indicated clade it was marked with 1; when genes were absent from all organism of that clade it was marked with 0. For example, group 1 represents the case where 4,244 *S. mansoni *genes were present in all three clades studied, and therefore no loss/gain events were computed; similarly, group 2, represents the case where 427 *S. mansoni *genes were not present in Nematodes or Arthropods, and under the APC hypothesis two loss/gain events per gene were computed, whereas under the LED hypothesis only one event per gene was counted. A schematic representation of these cases and the minimal number of events required under either of the two hypotheses is shown in Additional file 1. ** P-value was calculated using 100,000 bootstrapped samples followed by Wilcoxon Test.

We were primarily interested in the 427 genes from Group 2, i.e. genes conserved in schistosomes and deuterostomes, but absent in arthropods and nematodes (Table 1 and Additional file 1). Our rationale is that representative genes from this set, being absent in arthropods and nematodes, provide insights into platyhelminth-specific biology. The complete list of 427 genes in Group 2 can be found in Additional file 2. These genes were additionally submitted to manual inspection and comparison to gene data from the free-living non-parasite platyhelminth *S. mediterranea *(a planarian). The *S. mediterranea *gene collection [12] is the only other major set of platyhelminth data currently available.

We have found that 299 (70%) of the 427 *S. mansoni *genes (Additional file 2) are not present in the *S. mediterranea *EST [12] and genomic sequence datasets. It is difficult to evaluate which proportion of these 299 genes are actually present in *S. mediterranea *but perhaps not represented in this planarian partial sequence dataset. Further detailed investigation is still needed to confirm their absence in *S. mediterranea*.

The remaining 128 genes (30%) from Group 2 are shared with *S. mediterranea *(Additional File 2). These genes may play roles in platyhelminth-essential processes such as long-term tissue maintenance and cell turnover that are likely less important for short-lived organisms such as nematodes and insects. The list includes genes from pathways related to egg production, such as synthesis of mevalonate, as will be discussed later. Egg deposition is an essential step in the platyhelminth life-cycle and is the major cause of morbidity in the human host.

Out of the 427 genes in Group 2 we have selected three for further extensive investigation, characterizing their full-length sequence and pattern of expression, as described next. All three genes are also present in *S. mediterranea*; we believe that their putative roles in host interaction and signaling warrant special attention.

### Insulin Induced Gene (INSIG)

Among genes in Group 2, one of the most interesting is a *S. mansoni *INSIG ortholog (SmINSIG), an important regulator of the mevalonate synthesis pathway.

INSIG-1 in cultured mammalian CHO cells has been shown to play an essential role in degradation of HMG-CoA reductase (a critical enzyme in the mevalonate pathway) [23]. In *S. mansoni*, HMG-CoA reductase is vital for parasite survival and plays a physiological role in regulating egg production [24,25]. Egg deposition is a characteristic of platyhelminths, and in the case of *S. mansoni *the parasite's eggs deposited in the host circulatory system are the major cause of morbidity in the host's liver.

The full-length message of SmINSIG was obtained by RT-PCR. The reverse primer for RT-PCR was designed from the 3' end of a consensus sequence obtained by the *S. mansoni *Assembled EST designated SmAE C601385.1 and annotated as INSIG [9]. The forward primer was designed from a genomic region 119 bp upstream from the *locus *where the 5' end of SmAE C601385.1 is mapped; the genomic sequence can be found in Supercontig_0000071 of the *S. mansoni *genome draft sequence [11] publicly available at the Wellcome Trust Sanger Institute [26]. After cloning and sequencing the RT-PCR product we obtained the full-length SmINSIG message that contains 857 bases and encodes a protein of 244 amino acids. The deduced protein displays the described conserved domain of INSIG proteins (IPR009904) and the characteristic six transmembrane regions.

**Figure 2:**
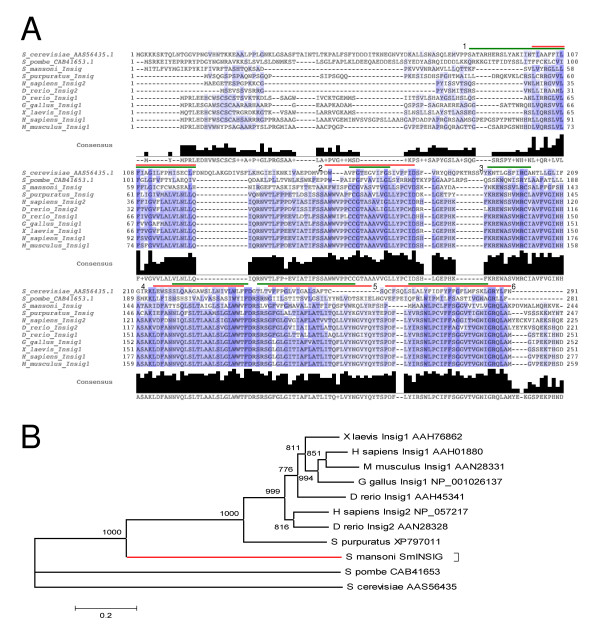
***S. mansoni *Insulin Induced Gene (SmINSIG) and its orthologs**. **A**: Multiple sequence alignment (MSA) of SmINSIG and several orthologs. Two distantly related yeast INSIG homologs were included in the MSA. SmINSIG transmembrane regions predicted by TMHMM and MINNOU are indicated by red and green bars, respectively. A consensus sequence and conservation bars are also represented; **B**: Maximum Likelihood tree constructed from the alignment of SmINSIG and several INSIGs found in public databases. The *S. mansoni *branch is represented in red. Numbers next to the branches represent bootstrap values (in 1000 samplings).

Figure 2A illustrates a multiple alignment of INSIGs from different deuterostomes and SmINSIG, where the conserved domain is shown and the six transmembrane regions are marked. BLASTP search against the nr database at GenBank using SmINSIG as query resulted in a best match to the zebrafish (*Danio rerio*) INSIG-1 ortholog ([GenBank: AAH45341.1]) with 49% identity and 68% similarity over 144 amino acids. A Maximum Likelihood tree was constructed (refer to the Methods section for details) (Figure 2B), and suggests that SmINSIG diverged before the gene duplication event responsible for emergence of the two vertebrate paralogs in zebrafish.

**Figure 3:**
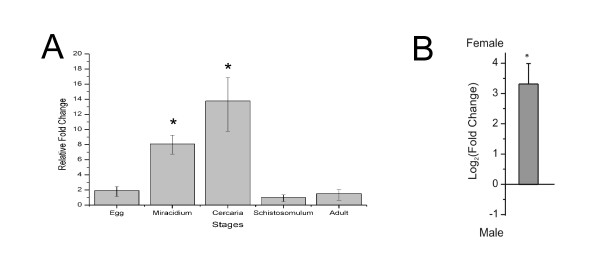
***S. mansoni *Insulin Induced Gene (SmINSIG) expression along the life cycle**. **A**: Real-time RT-PCR using total RNA samples from egg, miracidium, cercaria, schistosomulum or adult and primers for SmINSIG. Relative fold change was calculated by comparing the Ct value for each sample to Ct values for alpha-tubulin (internal standard). **B**: Real-time RT-PCR using mRNA samples from male and female adult worms and primers for SmINSIG. Log_2 _(Fold change) was calculated for the ratio between female and male expression values. *p < 0.05.

Real-time PCR experiments with tubulin as an internal standard showed a higher expression in egg, miracidium and cercarial stages when compared to schistosomulum (p < 0.05). The free-living forms (cercaria and miracidium) exhibit higher expression when compared to the forms that live inside the vertebrate host (schistosomulum and adult) (p < 0.05) (Figure 3A).

Despite previous studies showing induction of INSIG gene expression by insulin in rat regenerating liver [27] and the presence of genes encoding proteins with similarity to the human insulin receptor in schistosomes [9,28], we found that exposure of *S. mansoni *adult worms to exogenous recombinant human insulin peptide for 1 or 2 hours *in vitro *did not affect the expression of SmINSIG measured by Real-time RT-PCR while affecting the expression of a number of non-related genes (data not shown).

### Possible gene partners and targets of SmINSIG identified in schistosomes

This work is the first published report of an INSIG ortholog in a protostome invertebrate. We have searched the *S. mansoni *and *S. japonicum *transcriptome datasets for evidence of the genes described in vertebrates as targets of INSIG, and also for the presence of homologs in the mevalonate pathway. They are listed in Table 2 and are discussed in detail below.

**Table 2:** Putative SmINSIG interaction partners or possible downstream pathway genes in schistosomes, according to the INSIG functions and pathways described in the literature for other organisms

**Protein/gene**	**Accession [species]**	**Function**
Acetyl-CoA acetyltransferase	[GenBank: AAX26758.2]/[GenBank: AAX30459.1] [*S. japonicum*] C611014.1* [*S. mansoni*]	Mevalonate pathway
HMG-CoA reductase	[Swissprot: P16237] [*S. mansoni*]	Mevalonate pathway
Mevalonate kinase	C608152.1* [*S. mansoni*]	Mevalonate pathway
P-Mevalonate kinase	[GenBank: AAW26333.1] [*S. japonicum*] C608797.1* [*S. mansoni*]	Mevalonate pathway
Mevalonate diphosphate decarboxylase	C608650.1* [*S. mansoni*]	Mevalonate pathway
IPP isomerase	[GenBank: AAX26888.1] [*S. japonicum*]	Mevalonate pathway
gp78 Autocrine motility factor receptor (AMF receptor)	C609954.1*/C608624.1* [*S. mansoni*]	HMG-CoA reductase degradation
SCAP	C714360.1* [*S. mansoni*]	SREBP processing
Acetyl-CoA synthetase	C718554.1* [*S. mansoni*] [GenBank: AAX27415.2] [*S. japonicum*]	Fatty acid synthesis
Acetyl/Propionyl-CoA carboxylase	C611533.1* [*S. mansoni*] [GenBank: AAX27462.2] [*S. japonicum*]	Fatty acid synthesis

*These *S. mansoni *Assembled EST contigs are accessible at our *Schistosoma mansoni *EST Genome Project website [63] and contain publicly available GenBank EST sequences from [9].

Schistosomes are described to be cholesterol auxotrophs (unable to synthesize cholesterol) [29], suggesting that SmINSIG might perform in *S. mansoni *some of the functions described in mammals for INSIGs, especially in the mevalonate synthesis pathway (a precursor of cholesterol as well as of non-sterol isoprenoids) and not in the later steps of cholesterol synthesis. Accordingly, gene sequences potentially encoding all enzymes of the mevalonate pathway described in the MetaCyc database [30] were found either in *S. mansoni *or in *S. japonicum *public datasets, indicating that this pathway is intact in schistosomes (Table 2).

Interestingly, INSIG-1 has been shown to play an essential role in sterol-mediated degradation of HMG-CoA reductase (a critical enzyme in the mevalonate pathway) in cultured mammalian CHO cells [23]. Knockout mice have been generated in which the INSIG-1 and INSIG-2 genes are disrupted in the liver through recombination [31]. These mice have uncontrolled synthesis of fatty acids, cholesterol and a marked increase in HMG-CoA reductase protein, resulting from a decreased degradation of the reductase in the absence of INSIGs [31]. HMG-CoA reductase has an ortholog already characterized in *S. mansoni *[32] and its inhibition compromises egg production, as well as the survival of early stages of the parasite [24]. In addition, HMG-CoA reductase inhibition in schistosomes blocks egg production, which is induced by cholesterol precursors, such as mevalonate and farnesol; these precursors can also reverse the mevinolin-induced inhibition of egg production [25]. When expression was compared between male and female adult worms, a 10-fold higher expression of SmINSIG was found in females (Figure 3B). Egg production in female worms is a very efficient process and a putative negative control exerted by SmINSIG could be part of a modulatory mechanism of mevalonate synthesis in order to tune it to the rate of egg production. Miracidia and cercariae (larval forms) are short-lived and highly specialized forms, and the high levels of SmINSIG (Figure 3A) and the resulting inhibition of mevalonate synthesis in those stages when compared to adults would be consistent with a more complex physiology of the latter.

A recent report showed that in mammalian cells a fraction of INSIG-1 molecules are bound constitutively to a protein named gp78 or autocrine motility factor receptor (also present in schistosomes, see Table 2) through binding of transmembrane domains of the two proteins [33]. Evidence indicates that this sterol-triggered reductase/INSIG-1/gp78 complex is essential for ubiquitination and degradation of the reductase [33]. Based on the presence of orthologs in the *S. mansoni *transcriptome database, this evolutionarily conserved complex could also occur in schistosomes.

In line with the known *S. mansoni *auxotrophy for cholesterol, we could not identify either in the *S. mansoni *or in the *S. japonicum *transcriptomes any of the enzymes specifically involved in cholesterol synthesis from mevalonate. Interestingly, we found a *S. mansoni *EST with similarity to a short partial segment of *M. musculus *SCAP ortholog (Table 2); SCAP has been described in mammals as a SREBP cleavage-activating protein [34]. However, we found no gene fragments that would encode SCAP's putative partner SREBP, the Sterol Regulatory Element Binding Protein involved in regulation of cholesterol biosynthesis in mammalian cells. In the absence of any evident SREBP or cholesterol synthesis enzymes it is likely that the putative protein with partial similarity to SCAP might have a different role in *S. mansoni*.

Interfering with the mevalonate pathway in schistosomes could be a promising novel drug target approach because of the known adverse clinical consequences of eggs to the patients and of the biological importance of egg deposition in the parasite's life cycle.

### Vasohibin

Another gene from Group 2 that we have selected for further scrutiny is represented by *S. mansoni *Assembled EST (SmAE) C605907.1. Its consensus sequence had good (56%) similarity to vasohibin-like proteins (recently named vasohibin 2).

**Figure 4:**
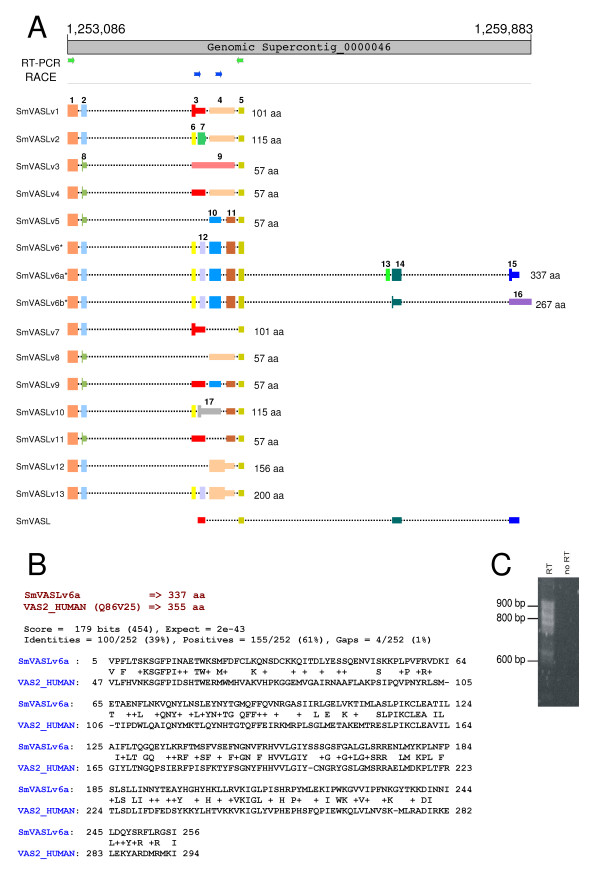
***S. mansoni *Vasohibin-like (SmVASL) isoforms**. **A**: Schematic representation of SmVASL transcripts aligned to the *S. mansoni *genomic sequence. The thick gray bar at the top represents the genomic sequence of Supercontig_0000046. Coding sequences, UTRs and introns are represented by thick, thin and dashed lines, respectively. We have colored and numbered the different exons consecutively in an arbitrary way, in the order that each new exon splicing form appears in Figure 4A. Primers that were used for the RT-PCR amplification and RACE experiments of SmVASL alternatively spliced forms are represented by green and blue arrows, respectively. Deduced protein-coding ORFs of SmVASL message are represented by thick colored lines, and the lengths of the deduced encoded proteins are displayed at the right side of each splice variant. The asterisks next to SmVASLv6, SmVASLv6a and SmVASLv6b indicate that the latter two are the result of a 3'-RACE-PCR experiment (3'-RACE primers represented by blue arrows); **B**: Local alignment (BLAST) showing the conserved region of SmVASLv6a and human vasohibin 2; **C**: Agarose gel electrophoresis of RT-PCR products from a reaction performed with primers indicated in panel A with green arrows. **RT **indicates that cDNA was synthesized by Reverse Transcriptase with poly-dT priming using RNA as template, and PCR was subsequently performed. **No RT **indicates a negative control where PCR was performed with an RNA sample incubated with poly-dT but no reverse transcriptase, to control for the absence of genomic DNA contamination.

Vasohibins constitute a recently described family of endothelium-derived angiogenesis inhibitors in humans [35,36]. The presence of a Vasohibin ortholog in schistosomes might suggest a potential angiogenesis inhibition process in the host, mediated by schistosomes' molecules. Complete sequencing of clone MA3-9999U-M294-F04-U.B, the longest clone in SmAE cluster C605907.1, showed a transcript with 975 bases (Figure 4A) that we named SmVASL for *S*. *mansoni *Vasohibin-like gene. BLASTP comparison to Swiss-Prot/TrEMBL showed the best match (48% identity and 58% similarity over 90 amino acids) to vasohibin 2 ([Swissprot: Q86V25] or FLJ12505 protein), a recently described human vasohibin-like protein [36]. The amino portion of SmVASL deduced protein (starting at Leu^4^) did align to Leu^142 ^of human vasohibin 2, thus suggesting that SmVASL represented a partial sequence of the *S. mansoni *ortholog.

To search for possible additional portions of SmVASL, the sequence of the original clone was mapped to the draft sequence of the *S. mansoni *genome [11]. We found that SmVASL maps with splicing to Supercontig _0000046, from base 1,255,010 to 1,259,701 (Figure 4A). In a genomic region of ~2,000 bases upstream from the SmVASL gene we found a sequence apparently encoding a peptide that displays similarity to vasohibin-like proteins (not shown). Next, we designed a forward and a reverse primer based on the sequence of this region, as indicated in Figure 4A (green arrows). These primers were used in a RT-PCR reaction, which resulted in a complex profile of amplification with one band of ~650 bp and a smear between 750–900 bp (Figure 4C). No amplicon was obtained in a parallel reaction without reverse transcription, to control for the absence of DNA contamination, indicating that the diverse range of products (Figure 4C) was derived from *bona fide *mRNA messages. This result suggested multiple alternatively spliced forms at the 5' end of full-length SmVASL.

The RT-PCR amplification products were cloned and sequenced. A total of approximately 300 clones were sequenced from both ends, disclosing fourteen different isoforms (SmVASLv1-13, with two SmVASLv6 isoforms). Mapping of these isoforms to the *S. mansoni *genomic sequence confirmed that the different clones are a result of alternative splicing (Figure 4A). Variation is caused by a number of alternative splicing events, such as 5'-deletion, exon skipping, and junction of two exons by intron retention. Two different lengths for the second exon were observed: exon 2 with 79 bp for variants SmVASLv1, 2, 6a, 6b, 7, 10, 12 and 13; and exon 8 with 65 bp, resulting from a 5'-deletion in exon 2, for SmVASLv3, 4, 5, 8, 9 and 11 (Figure 4A). The latter splicing form caused an early stop, and the resulting isoforms encode a putative short 57 amino acid protein. The isoforms have different 3'-UTR ends, which may be related to different stability of the messages.

Exon skipping was observed in SmVASLv5, 7, 8, 11 and 12 (Figure 4A). Intron retention was detected in isoforms SmVASLv1, 2, 4, 12 and 13, where exon 4 is the junction of exons 10 and 11 (that are present for example in isoform 5). Intron retention was also detected in SmVASLv1, 4, 7, 9 and 11, where exon 3 is the junction of exons 6 and 7. Finally, the intron retention observed in SmVASLv3 results from junction of exons 6, 7, 10 and 11.

**Figure 5:**
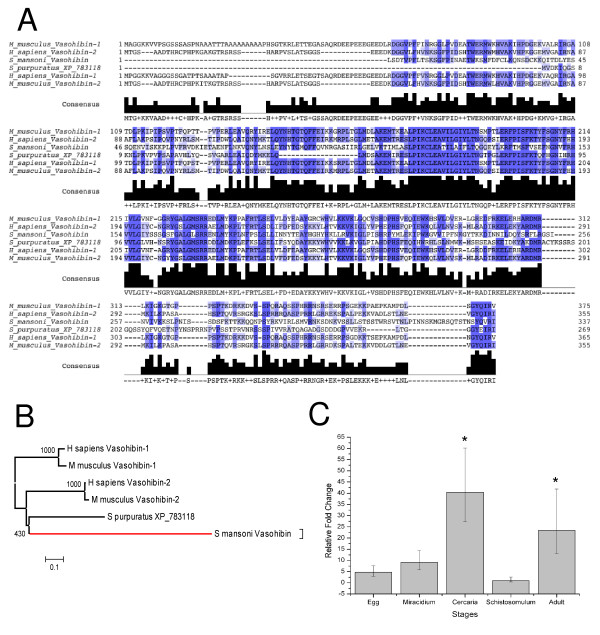
***S. mansoni *Vasohibin orthologs and expression along the life cycle**. **A**: Multiple sequence alignment of SmVASLv6a (the longer isoform) and several orthologs. **B**: Maximum Likelihood tree constructed from the alignment of SmVASLv6a and other vasohibins found in public databases. The *S. mansoni *branch is represented in red. Numbers next to the branches represent bootstrap values (in 1000 samplings). **C**: Real time RT-PCR using total RNA samples from egg, miracidium, cercaria, schistosomulum or adult and primers for SmVASL. Relative fold change was calculated by comparing the Ct value for each sample to Ct values for alpha-tubulin (internal standard).

We have identified seven different polypeptides that are encoded by these variants, representing different deduced protein isoforms (Figure 4A). As noted earlier, isoforms SmVASLv3, 4, 5, 8, 9 and 11 encode the same 57 amino acid protein. Due to the absence of a stop codon in the SmVASLv6 sequence in the segment amplified by RT-PCR, a 3'-RACE experiment was conducted to extend SmVASLv6 sequence, which resulted in two additional longer SmVASLv6 isoforms, SmVASLv6a and SmVASLv6b. The longer isoform, SmVASLv6a encodes a 337 amino acid protein, the longest protein isoform detected here. When compared through BLASTP with the Swiss-Prot/TrEMBL database, the highest match was a protein of *S. japonicum *of unknown function ([Swissprot: Q5DB91]). The second best match was to human vasohibin-like protein (vasohibin 2, [Swissprot: Q86V25]) (Figure 4B) to which SmVASLv6a protein aligns with 37% identity and 57% similarity over 252 amino acids (71% coverage of the human protein). Alignment of SmVASLv6a and human vasohibin 2 reveals a C-terminal divergence between these two proteins (Figure 4B). 5'-RACE experiments under several conditions were performed, without success. A multiple sequence alignment and a phylogenetic tree of SmVASLv6a and several other orthologs are represented in Figure 5A and 5B, respectively.

Interestingly, several alternatively spliced isoforms are predicted for human vasohibin 2 ([Swissprot: Q86V25]), some of them encoding a shorter protein that lacks the carboxyl-terminal end. Similarly, all SmVASL but SmVASLv6a and SmVASL6b isoforms encode shorter proteins, lacking different portions of the carboxyl-terminal end. Absence of the C-terminal end has been also described for one of the isoforms of vasohibin 1, where the shorter isoform abolishes the anti-angiogenic activity of the full-length protein [35]. Conservation of such a mechanism for generation of short isoforms lacking the carboxyl-terminal region of the protein in distantly related species such as *H. sapiens *and *S. mansoni *indicates that the C-terminal portion of vasohibin should represent a conserved functional domain.

Real-time PCR experiments using primers designed to detect most of the isoforms (except isoform 3), shows that cercaria and adult have the highest SmVASL expression levels, whereas schistosomulum has the lowest SmVASL levels (Figure 5C). Considering the angiogenesis control role exerted by vasohibin in vertebrates and the high conservation level between SmVASL and human vasohibin, together with vascular location of adult schistosomes, it is tempting to hypothesize a role of SmVASL in modulating human angiogenesis. Considering the presence of a vasohibin homolog in planarian (Additional file 2) and the high SmVASL expression in miracidium, the *S. mansoni *life form that invades the snail (an invertebrate host that has an open circulatory system), we speculate that this protein could perform a different (possibly endogenous) role in this life stage. Loeffler et al. [37] reported that schistosomes exert a positive effect on angiogenesis because soluble egg antigen (SEA) induces angiogenesis-related processes by up-regulating VEGF in human endothelial cells [37]. It is hypothesized that neovascularization in the schistosome granuloma may be necessary to maintain oxygen and nutrient levels, in a similar way as in rapidly growing tumors [37,38]. Negative regulation of host angiogenesis may be provided by the *S. mansoni *well conserved vasohibin ortholog identified here, which would counterbalance the previously reported positive effects of *S. mansoni *on angiogenesis [37], thus helping to maintain host hemostasis. Functional experiments are warranted to determine the biological roles of the SmVASL isoforms reported here.

### Interferon Regulatory factor

The third and final gene analyzed in the present study encodes an Interferon Regulatory Factor (IRF) protein (represented by SmAE C603512.1 and named SmIRF).

**Figure 6:**
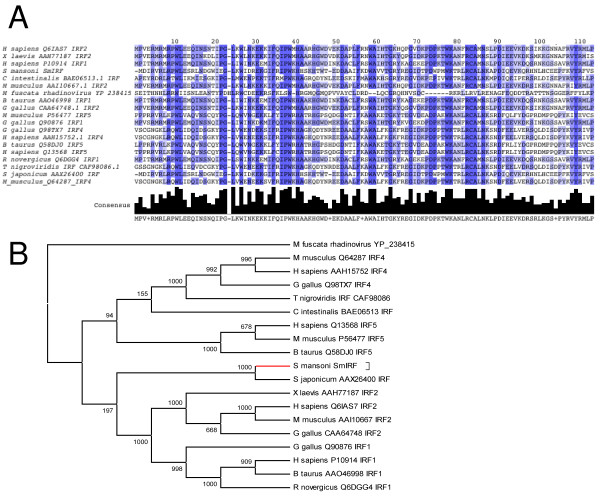
***S. mansoni *Interferon Regulatory Factor (SmIRF)**. **A**: Multiple sequence alignment of SmIRF and several orthologs. For displaying purposes, only the conserved region is represented; **B**: Maximum Likelihood tree constructed from the alignment of SmIRF and several IRFs found in public databases. The *S. mansoni *branch is represented in red. Numbers next to the branches represent bootstrap values (in 1000 samplings).

IRFs are important molecules involved in immune response and a schistosome ortholog could be related to the recognition and response to host immune processes. Primers were designed from the extremities of this assembled sequence and a single 1330 bp amplicon was generated by RT-PCR; cloning and sequencing confirmed its identity. In addition, a 3'-RACE with primers designed from SmAE C603512.1 permitted cloning and sequencing of the 3' end of this mRNA. SmIRF full-length sequence has 2297 bp and encodes a 476 amino acid protein that shares 27% identity and 44% similarity over 434 amino acids with the interferon regulatory factor 4 of *Gallus gallus *([GenBank: AAK08198]) [see Additional file 3]. A multiple sequence alignment and a phylogenetic tree of SmIRF and several other orthologs are represented in Figure 6A and 6B, respectively. Each IRF contains a well-conserved DNA-binding domain with ~120 amino acids at the amino terminus that folds into a helix-turn-helix DNA-binding motif (Smart SM00348). The C-terminal end of IRFs is generally more variable among family members [39] and contains a SMAD/FHA domain, commonly found in transcription factors and responsible for interaction with other phosphorylated molecules [40]. *In silico *analysis of SmIRF using NetPhos [41] revealed the presence of several serine phosphorylation sites [Additional file 3]; in mammalians, IRF phosphorylation is important for its function and regulation [42].

IRFs constitute a complex family of transcription factors with broad functionality in mammalians, which was recently reviewed [43]. Honda and Taniguchi [43] pointed to several mammalian genes regulated by IRFs and we have searched for orthologs of these targets in *S. mansoni*. Two genes regulated by IRFs (TAP1 and LMP2), involved in processing and transport of peptides appear to have homologs in schistosomes. Using human TAP1 gene encoded protein as query we found in *S. mansoni *an ABC transporter gene, SMDR1 [GenBank: AAA66476.1] (35% identity and 61% coverage) that has been previously described as possible transporter of peptides [44]. When using human LMP2 ([GenBank: CAA60784]) as query, we found a schistosome ortholog in our database (C602473.1, with 55% identity and 88% coverage). Human LMP2 encodes a proteasome subunit and replaces the Y subunit in the proteasome structure upon IFN signaling and this change results in a different proteasome proteolytic activity [45]. Functional proteasomes were recently shown to be essential for schistosome development in the vertebrate host [46]. IRFs also regulate caspase-1 expression [43]. We found a caspase in *S. mansoni *that has higher similarity with caspase-3 than with caspase-1 (44% identity and 85% coverage); in mammalians both caspases are involved with apoptosis control; however, the apoptosis pathway appears to be incomplete in the parasite [9].

Besides the inherent capacity of IRFs to function as transcription factors, they selectively bind to a group of proteins, namely immunophilins. Interaction between IRF-4 and immunophilin FKBP52 in mammalians results in a conformational change, abolishing DNA-binding activity and partial IRF-4 proteolysis [47]. One immunophilin named p50 [GenBank: AAA69867.1] was already identified in schistosomes, having a high similarity with vertebrate FKBP immunophilins [48,49].

The IRF interactions are highly complex and not fully elucidated, even in mammalians, with new interactions and functionalities being recently reported [43]. In this work we have pointed to the existence in schistosomes of some possible players. Other experiments (e.g. protein-protein interaction) are warranted to identify functions and other possible SmIRF interaction partners. It would be interesting to demonstrate if SmIRF is involved in schistosome stress responses (viral infections, for example), or in host-parasite interaction, being part of the pathway that interplays with host immune molecules.

## Conclusion

According to our analysis, the *S. mansoni *genes described in this work are homologs of genes present in Deuterostomia, but absent in Ecdysozoa organisms. Given the specific functions of these genes in Deuterostomia, especially in mammals, we envisage the possibility of co-optation of the schistosome ortholog in interaction and adaptation to the host environment. The evolutionary history leading to conservation of such set of genes in these two distantly related groups is not totally clear yet; more sequence information from organisms of related phyla (especially from other Lophotrocozoa) should help throw light on this problem. The three genes characterized in detail in this work might be involved in mevalonate synthesis (egg production), angiogenesis control (host invasion) and immune response (interplay with host defense molecules).

In *S. mediterranea *the orthologs of these genes may perform ancestral non-parasitic roles. In this respect, a recent report [50] revealed the presence of orthologs of thyroid hormone receptor (TR) in *S. mansoni, S. japonicum *and *S. mediterranea *and demonstrated that TRs in platyhelminths are highly conserved not only in sequence similarity, but also in gene organization, protein-protein interaction and in DNA-binding ability [50]; TR was previously believed to be an innovation of chordates as the genomes of insects and nematodes do not contain TR genes. Although the functions of TRs in invertebrates are not fully understood, phylogenetic analysis showed that the TR ortholog likely originated from a common ancestor of the Bilateria [50]. Both the results of Wu et al. [50] and ours suggest the presence in a common ancestor of genes previously thought to have arisen only later in evolution. We hypothesize that during *Schistosoma *evolution the three genes discussed in the present report might have been co-opted to perform functions that are crucial for the parasite-host interplay. Regardless of the actual history, we believe the genes discussed here have characteristics that make them good candidates for further investigation as potential drug targets.

## Methods

### Datasets

*S. mansoni *EST sequences generated by our group [9] were used as the query dataset of *S. mansoni *sequences in this work. Local BLAST [51] databases were formatted with all the sequences available from arthropods, nematodes and deuterostomes in the non redundant (nr) nucleotide section of GenBank database (updated as of May/2007) and were used as subject in BLAST searches. In addition, all the publicly available nucleotide sequences from non model arthropods, nematodes and from the planarian *S. mediterranea *were used. The planarian dataset we used is composed of all the publicly available 171,483 nucleotide sequences (97,901 CoreNucleotide and 73,582 ESTs, on May 2007) together with the assembled ESTs obtained by a large scale EST sequencing project [12]. The planarian dataset was further supplemented with the EST assembly data provided by the authors (10,485 assembled sequences; 6,488 contigs and 3,997 singlets) [12]. In order to reduce the EST sequence redundancy and consequently the time complexity of the process, the sequences were processed using CD-HIT [52] prior to database formatting. After identifying the *S. mansoni *genes of interest, we have considered their downstream effectors described in the literature for vertebrates and we searched for the presence of orthologs which could act as molecular partners or members of downstream pathways in *S. mansoni*. When any of these orthologs were missing in the *S. mansoni *GenBank database the *S. japonicum *sequences were alternatively searched.

The *S. mansoni *genome sequence partial assembly (v. 3) at the Wellcome Trust Sanger Institute [26] was used.

### Similarity searches

We used the BLASTX and TBLASTX programs [51] to search for *S. mansoni *(a platyhelminth) translated sequences that have considerable similarity to proteins and translated ESTs from deuterostomes, nematodes and arthropods. Perl scripts together with the Zerg parser [53] and BioPerl [54] were used to perform and process BLAST searches.

BLAST alignments with bit scores higher than 100 and lower than 50 were considered as true matches and non-significant hits, respectively. Bit scores were used to facilitate cross-database comparisons. Hits with intermediary bit scores (between 50 and 100) were manually inspected, basically to eliminate hits derived from unmasked low complexity regions where matches occur through a single amino acid at repeated intervals. Overall, the sequences included in Additional File 2 have scores higher than 80.

The *S. mansoni *genes without matches and *Schistosoma *specific genes were filtered out. The resulting genes were further assigned to one of the following groups (Table 1 and Additional file 1) when a given gene was detected in at least one species belonging to the corresponding clade: (1) present in *S. mansoni*, deuterostomes, arthropods and nematodes (all groups); (2) present in *S. mansoni *and deuterostomes, but not in arthropods and nematodes; (3) present in *S. mansoni *and arthropods, but not in deuterostomes and nematodes; (4) present in *S. mansoni *and nematodes, but not in arthropods and deuterostomes); (5) present in *S. mansoni*, arthropods and nematodes, but not in deuterostomes; (6) present in *S. mansoni*, arthropods and deuterostomes, but not in nematodes; (7) present in *S. mansoni*, nematodes and deuterostomes, but not in arthropods.

To evaluate the significance of the gene gains/losses under each hypothesis, we have generated 100,000 bootstrapped samples and performed the Wilcoxon test. Simulations and the significance test were performed in the R environment.

TMHMM [55] and MINNOU [56] were used to predict transmembrane domains, NetPhos [41] to identify possible phosphorylation sites, with SignalP 3.0 [57] to predict signal peptides, and with InterProScan [58] to predict conserved domains.

### Phylogenetic analysis

Multiple Sequence Alignments in the paper were generated using MUSCLE [59] and edited using JalView [60]. Curated alignments were then used to generate Maximum Likelihood phylogenetic trees with PhyML package [61]. Significance of the results was estimated by building 1000 bootstrapped trees. The NEWICK files generated by PhyML were then displayed with MEGA [62] producing the trees displayed in the results section.

### Cloning procedures

mRNA was obtained from adult parasites conserved in RNALater (Ambion, Austin, TX, USA) by extraction of tissue with MACs mRNA isolation kits (Miltenyi Biotec, Bergisch Gladbach, Germany). 200 ng of mRNA were treated with 5 U of RQ1 RNAse-free DNAse (Promega, Madison, WI, USA) for 30 min at 37°C. Reverse transcription was performed with oligo dT primers using the protocol of Superscript first strand system for RT-PCR (Invitrogen, Carlsbad, CA, USA). PCR was performed with Advantage II (BD Biosciences, Palo Alto, CA, USA) with the buffer supplied by the manufacturer, and 200 nM of each specific primer using the following cycling program: 95°C for 1 min plus 35 cycles each at 95°C for 30 s, 55°C for 30 s, and 68°C for 3 min, followed by a final extension at 68°C for 3 min. The products were analyzed in 1.2% agarose gel and cloned in pGEM-T vector (Promega) for further sequencing. Primers are listed in Additional file 4.

### Rapid amplification of cDNA ends (RACE)

mRNA was obtained from adult parasites conserved in RNALater (Ambion) by extraction of tissue with MACs mRNA isolation kits (Miltenyi Biotec). 200 ng of mRNA was used for reverse transcription using the protocol of the 3'RACE system kit for rapid amplification of cDNA ends (Invitrogen) and specific primers. To perform 3' RACE of vasohibin gene, 3 μg of total RNA from miracidium was used. Reverse transcription was performed using 1 μl of Super Scrip III (Invitrogen) at 65°C for 5 min, 55°C for 50 min and 85°C for 5 min.

PCR reaction was performed with Advantage II polymerase (BD Biosciences) with buffer supplied by the manufacturer, 200 μM dNTPs and 200 nM of each primer using the following cycling program: 95°C for 1 min plus 35 cycles each at 95°C for 30 s, 55°C for 30 s, 68°C for 3 min, followed by a final extension at 68°C for 3 min. The products were analyzed in 1.2% agarose gel and cloned in pGem-T vector (Promega) for further sequencing. Primers are listed in Additional file 4.

### Real-time RT-PCR procedures

mRNA was obtained from male or female adult parasites conserved in RNALater (Ambion) by extraction of tissue with MACs mRNA isolation kits (Miltenyi Biotec). 200 ng of mRNA were treated with RQ1 RNAse-free DNAse (Promega), using 1 U in 10 μl of reaction, for 30 min at 37°C. The DNAse was inactivated at 65°C for 10 min.

Three micrograms of total RNA from each of five stages was treated with RQ1 RNAse-free DNAse (Promega), using 4 U in 10 μl of reaction, for 1 hour at 37°C. The resulting products were reverse transcribed with random hexamer primers using the protocol of Superscript first strand system for RT-PCR (Invitrogen). Control reactions without addition of reverse transcriptase were run in parallel, and were used as templates for PCR negative control, to control for the absence of possible DNA contaminants. Primers for real-time RT-PCR (listed in Additional file 4) were designed using the Primer Express program version 2.0.0 (Applied Biosystems, Foster City, CA, USA) with default parameters. Real-time RT-PCR reactions were performed using SYBR Green PCR master mix (Applied Biosystems) and the specific primers in a GenAmp 5700 Sequence Detection System (Applied Biosystems). P-value was calculated using Student's t-test with 95% confidence interval.

### EMBL sequence deposition

All sequences determined in this work were deposited at EMBL under the following numbers: SmINSIG, [EMBL: AM493258]; SmIRF, [EMBL: AM493259]; SmVASL isoforms, [EMBL: AM493260 – AM493273].

## Authors' contributions

TMV conceived the study and carried out the computational analysis. RdeM, GTA and KCP performed the wet-lab experiments. TMV and RdeM analyzed the data. TMV, RdeM, JCS and SVA participated in the discussion of results and drafting of the manuscript. SVA coordinated and supervised the project. All authors read and approved the final manuscript.

## Supplementary Material

Additional file 1 Schematic representation of the relationships between *S. mansoni *and three different clades and indication of the several groups that result from the presence or absence of *S. mansoni *genes among the organisms of each of the three clades. Click here for file

Additional file 2 List of group 2 genes (conserved in schistosomes and deuterostomes, but lost in nematodes and arthropods). This table provides full statistics about query and hit coverage, identity and similarity percentages and annotations. Column "P" represents the presence (1) or absence (0) of a planarian homolog. The first four entries are for the three genes that were further characterized in this work. Click here for file

Additional file 3 **Conserved domains in SmIRF**. **A**: BLAST analysis against the SWISSPROT database. A conserved N-terminal DNA-binding domain in SmIRF can be easily detected. **B**: *In silico *analysis of SmIRF using NetPhos revealed the presence of several Serine phosphorylation sites, important for IRF function and regulation. Click here for file

Additional file 4 Primers used in the Real-time RT-PCR and RACE experiments. Click here for file
