# Supplementary material for: Analysis of Schistosoma mansoni genes shared with Deuterostomia and with possible roles in host interactions
Source: BMC Genomics. 2007 Nov 8;8:407. doi: 10.1186/1471-2164-8-407 (PMC2194728; doi:10.1186/1471-2164-8-407)
Supplement: Additional file 1 — Schematic representation of the relationships between S. mansoni and three different clades and indication of the several groups that result from the presence or absence of S. mansoni genes among the organisms of each of the three clades. [file 1471-2164-8-407-S1.pdf]

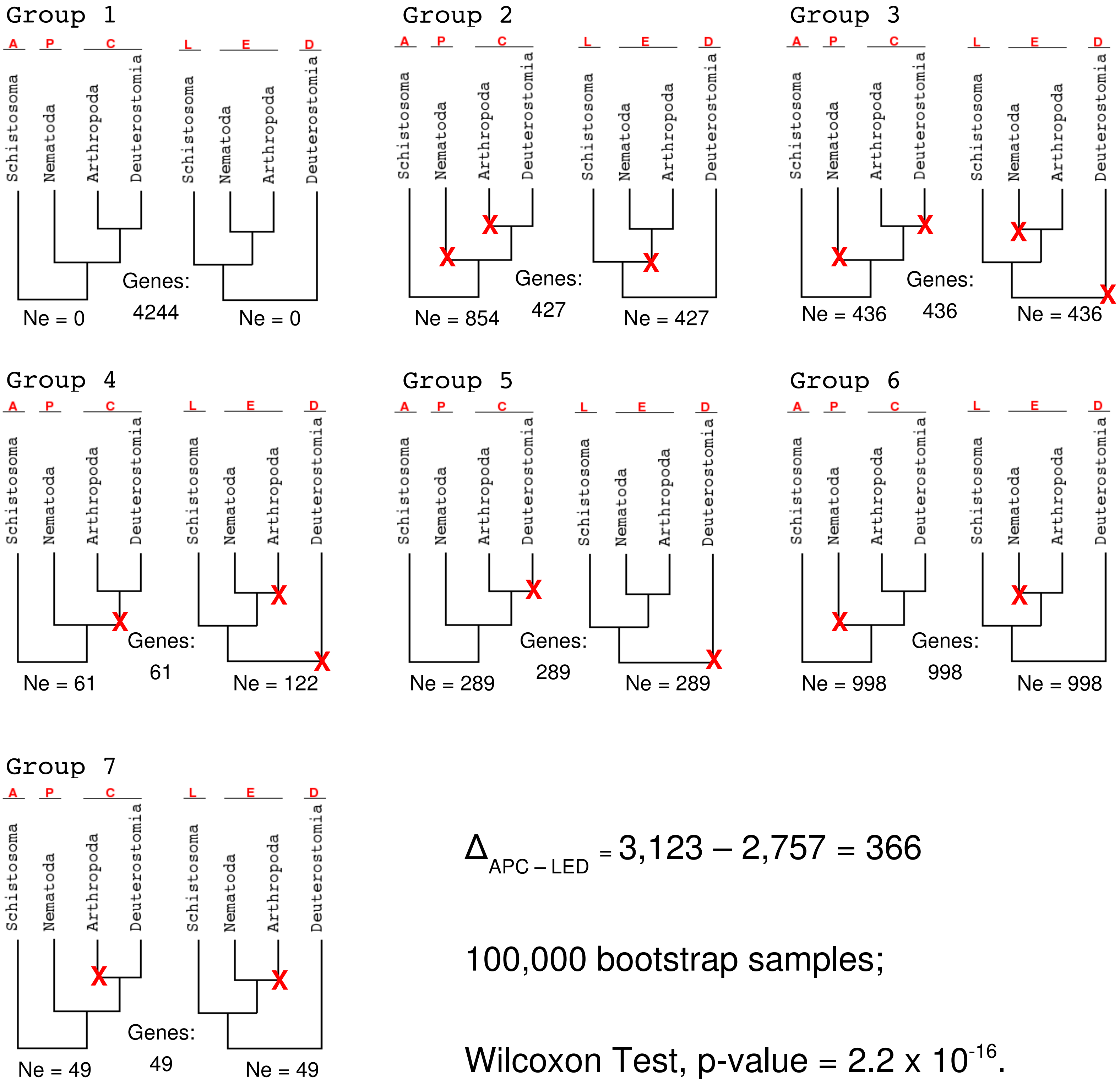

Additional file 1: Schematic representation of the relationships between S. mansoni and three different clades, and indication of the seven groups that result from the presence or absence of S. mansoni genes among the organisms of each of the three clades. The two main evolutionary hypotheses for the relationships between animal phyla are shown (APC or LED). The X's represent points of gene loss for the simplest set necessary to explain the presence/absence of a gene under the given model. "Genes:" shows the number of S. mansoni genes identified as present in each group; "Ne=" shows the number of loss/gain events for that group under the given evolutionary hypothesis.
