# Supplementary material for: Analysis of Schistosoma mansoni genes shared with Deuterostomia and with possible roles in host interactions
Source: BMC Genomics. 2007 Nov 8;8:407. doi: 10.1186/1471-2164-8-407 (PMC2194728; doi:10.1186/1471-2164-8-407)

Additional file 3: Conserved domains in SmIRF.

A: BLAST analysis against the SWISSPROT database. A conserved N-terminal DNA-binding domain in SmIRF can be easily detected. **B:** *In silico* analysis of SmIRF using NetPhos revealed the presence of several Serine phosphorylation sites, important for IRF function and regulation.

A

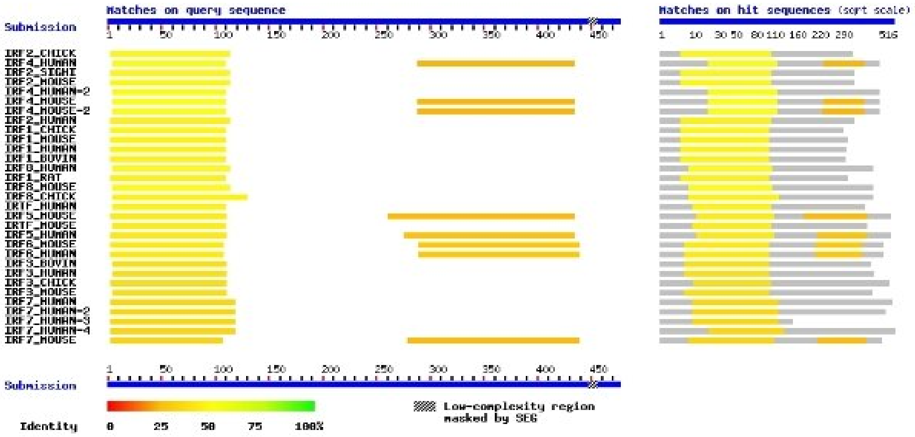

B

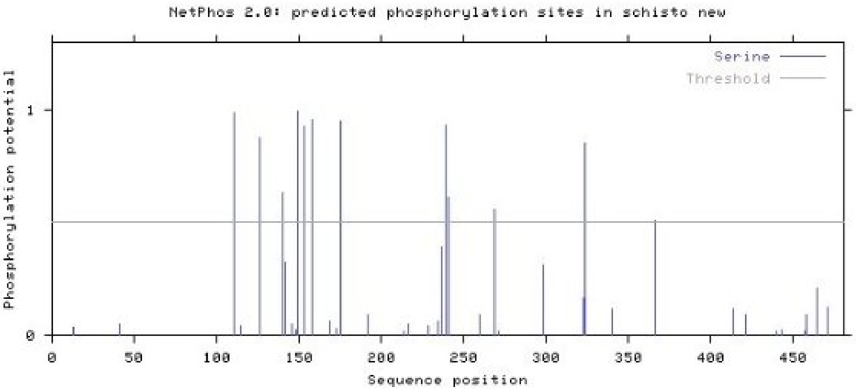

Supplement: Additional file 3 — Conserved domains in SmIRF. A: BLAST analysis against the SWISSPROT database. A conserved N-terminal DNA-binding domain in SmIRF can be easily detected. B: In silico analysis of SmIRF using NetPhos revealed the presence of several Serine phosphorylation sites, important for IRF function and regulation. [file 1471-2164-8-407-S3.pdf]
