# Supplementary material for: Analysis of Schistosoma mansoni genes shared with Deuterostomia and with possible roles in host interactions
Source: BMC Genomics. 2007 Nov 8;8:407. doi: 10.1186/1471-2164-8-407 (PMC2194728; doi:10.1186/1471-2164-8-407)
Supplement: Additional file 4 — Primers used in the Real-time RT-PCR and RACE experiments. [file 1471-2164-8-407-S4.pdf]

Additional file 4: Primers used in this study

Name	Primer sequence
3'-RACE GSP 1	ATTAGTAAAGATAGACTTGGAAAGTTAAGC
3'-RACE GSP 2	ATCTATTATTAGACTTGGGGAGCTTG
Vasohibin FW (real-time PCR)	AGCATATCATGGTCATTATCAC
Vasohibin RV (real-time PCR)	CAACATGTAAGGTCGATGTG
Tubulin FW	CCATTTATGATATTTGTCGACGGA
Tubulin RV	TTTGTGTAGGTTGGACGCTCTATATCTA
INSIG FW	CAGGACAATGACGACACCACC
INSIG RV	GCTGCTTGGTTGCCTTGTCT
